# URMylation Signaling Pathway in Cancer: Molecular Mechanisms and Therapeutic Opportunities

**DOI:** 10.3390/cimb48090869

**Published:** 2026-08-27

**Authors:** Jingchao Wang, Peiqiang Yan, Weiwei Jiang, Li Chen, Daoyuan Huang, Tao Hou, Hiroyuki Inuzuka, Wenyi Wei

**Affiliations:** Department of Pathology, Beth Israel Deaconess Medical Center, Harvard Medical School, Boston, MA 02215, USA; jwang32@bidmc.harvard.edu (J.W.); pyan2@bidmc.harvard.edu (P.Y.); wjiang2@bidmc.harvard.edu (W.J.); lchen24@bidmc.harvard.edu (L.C.); huang5@bidmc.harvard.edu (D.H.); thou@bidmc.harvard.edu (T.H.); hinuzuka@bidmc.harvard.edu (H.I.)

**Keywords:** URMylation, URM1, MOCS3, tRNA thiolation, oxidative stress, proteostasis, cancer therapy

## Abstract

Ubiquitin-related modifier 1 (URM1) defines a distinctive ubiquitin-like system in which URM1 functions both as a covalent protein modifier and a sulfur carrier required for wobble uridine (U34) thiolation of cytosolic tRNAs. This dual role places URM1 signaling at the intersection of protein post-translational modification and codon-sensitive translational control. Although URM1 remains less well characterized than ubiquitin and other ubiquitin-like modifiers, accumulating evidence links URM1-associated processes to oxidative-stress tolerance, proteostasis, and tumorigenesis. In this review, we summarize the molecular architecture and biochemical regulation of URM1 signaling, distinguish the biological significance of protein URMylation from URM1-dependent tRNA U34 thiolation, and evaluate evidence connecting these pathways to cancer biology. Notably, stress-inducible protein URMylation has been demonstrated in mammalian cells, although endogenous mammalian URM1 substrates and their cancer-relevant functions remain incompletely defined to date. In parallel, URM1-dependent tRNA thiolation has been linked to selective translational programs that support metastatic progression and adaptation to targeted therapy. We further discuss emerging therapeutic opportunities associated with redox and translational dependencies, as well as the potential limitations and challenges of pathway selectivity, biomarker development, and normal-tissue toxicity. Together, these studies position URM1 signaling as a context-dependent stress-adaptive network with emerging relevance to cancer biology and precision oncology.

## 1. Introduction

Cancer cells survive through both stable molecular alterations and continuous adaptation to the surrounding environmental changes [1,2]. Oncogenic mutations, tumor-suppressor loss, chromosomal instability, and persistent oncogenic signaling shape tumor biology, but they do not fully explain how malignant cells tolerate the physiological stress imposed by uncontrolled cell growth [3,4,5]. In this context, tumor cells frequently encounter nutrient limitation, oxidative stress, immune pressure, metabolic imbalance, and therapy-induced injury, all of which require rapid regulatory responses beyond fixed genomic lesions [3,6]. Protein post-translational modification (PTM) systems contribute to this adaptive capacity by regulating protein abundance, enzymatic activity, localization, interaction networks, and signal duration [7,8]. In this regard, protein ubiquitination and deubiquitination processes are broadly implicated in cancer biology and therapeutic development [9]. In addition, other types of ubiquitin-like (UBL) signaling also contribute to tumorigenesis. For example, SUMOylation regulates transcriptional control, DNA-damage responses, and survival pathways [10], whereas neddylation is frequently dysregulated in malignancy and remains pharmacologically tractable [11]. ISGylation also contributes to tumor immunity and malignant phenotypes in a context-dependent manner [12], while UFMylation has been linked to proteostasis, stress responses, and tumorigenesis [13,14]. By contrast, URMylation remains less well characterized than these UBL pathways, particularly in the cancer setting.

URMylation is mediated by the ubiquitin-related modifier 1 (URM1), an evolutionarily conserved UBL protein with unusual biochemical properties [15]. URM1 contains a ubiquitin-like β-grasp fold and retains features that place it close to the evolutionary origin of protein modifiers [16]. Like other UBLs, URM1 has a C-terminal glycine that can be activated for covalent attachment to target protein substrates, and early work identified the yeast peroxiredoxin Ahp1 as a URM1-conjugated protein involved in oxidative-stress protection [17]. Subsequent studies showed that URM1 can form lysine-directed conjugates and that oxidative stress enhances the URMylation of target proteins in yeast and mammalian cells [18]. On the other hand, URM1 also acts as a sulfur carrier required for the thiolation of cytosolic tRNA wobble uridine, as shown by functional proteomics and biochemical studies [19,20]. This dual-functional role places URM1 signaling at the intersection of protein modification, sulfur transfer, tRNA chemistry, translational decoding, and cellular stress adaptation [15,21,22].

This dual biochemical identity also provides a rationale for considering URM1 signaling in cancer biology. Tumor cells often operate near the limits of redox and proteostatic capacity due to oncogene-driven biosynthesis, mitochondrial dysfunction, metabolic rewiring, and exposure to chemotherapy or targeted therapy [6,23,24]. Protein URMylation may influence the behavior or localization of selected stress-sensitive proteins, whereas URM1-dependent tRNA thiolation may affect decoding fidelity and the translation of selected protein networks, thereby impacting tumorigenesis [18,19,25,26]. Although direct cancer-focused studies of URMylation have remained limited to date, the pathway is positioned to influence processes central to malignant adaptation, including stress tolerance, proteome maintenance, translational control, and therapy response. This review therefore examines the molecular architecture and functional outputs of URM1 signaling, distinguishes covalent protein URMylation from URM1-dependent tRNA thiolation, and evaluates how these interconnected outputs may inform cancer biology and precision-oncology strategies.

## 2. Evolutionary Logic and Molecular Architecture of URM1 Signaling

URM1 signaling is best understood as an evolutionarily conserved system that preserves features of both ancient sulfur-transfer chemistry and eukaryotic ubiquitin-like modification. This dual identity explains why URM1 cannot be interpreted simply as another ubiquitin-like modifier and why its biological output must be considered in terms of both covalent protein conjugation and sulfur-dependent tRNA modification.

### 2.1. From Sulfur Carrier to Bifunctional UBL Signaling

**Figure 1:**
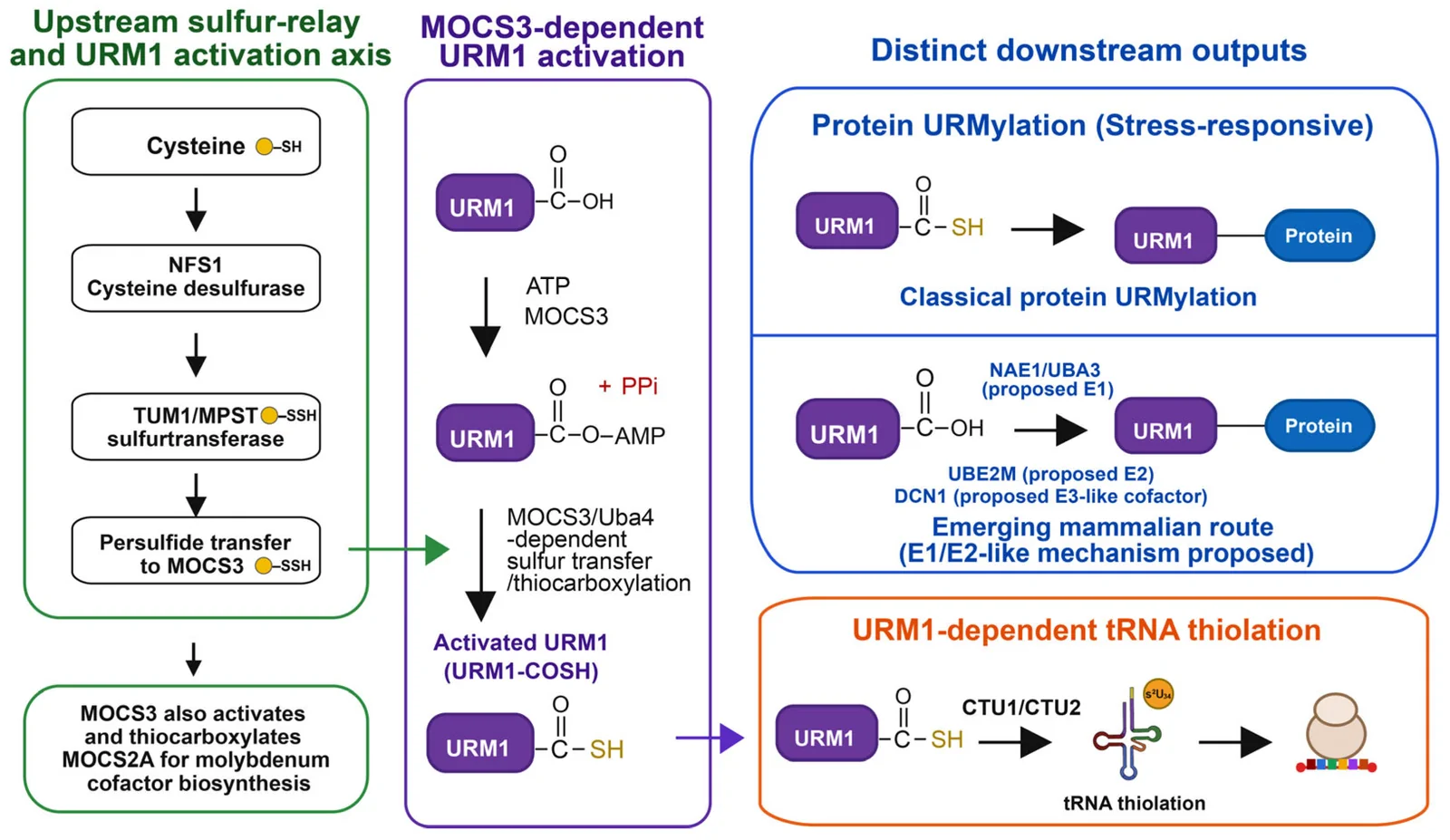
Molecular architecture and distinct outputs of URM1 signaling. Cysteine-derived sulfur is mobilized by NFS1 and relayed via the TUM1/MPST sulfur-transfer system toward MOCS3 (Uba4 in yeast), which then catalyzes ATP-dependent URM1 activation and thiocarboxylation to generate URM1-COSH. Thiocarboxylated URM1 supports both stress-responsive protein URMylation and CTU1/CTU2-dependent tRNA U34 thiolation. The classical MOCS3-dependent pathway is distinguished from the recently proposed NAE1/UBA3–UBE2M route for mammalian protein URMylation, with DCN1 proposed as an E3-like cofactor. MOCS3 also contributes to molybdenum cofactor biosynthesis through MOCS2A thiocarboxylation. Brownish-yellow circles in the left panel indicate sulfur transfer from cysteine through persulfide intermediates to MOCS3. The green arrow denotes sulfur transfer into MOCS3-dependent URM1 activation, whereas the purple arrow indicates downstream use of URM1-COSH in protein URMylation and CTU1/CTU2-dependent tRNA U34 thiolation.

URM1 entered the ubiquitin-like modifier field as an unusual small protein with features of both eukaryotic protein modifiers and ancient sulfur-carrier systems (Figure 1). Human URM1 is a small 101-amino-acid protein with a calculated molecular mass of approximately 11.4 kDa [27]. The first description of the yeast URM1 pathway identified a protein-conjugation system homologous to prokaryotic biosynthetic enzyme reactions, thereby distinguishing URM1 from canonical ubiquitin-like modifiers at the time of its discovery [28]. Subsequent studies showed that URM1 and its associated enzyme Uba4 participate in invasive growth and budding in yeast, indicating that this pathway has cellular functions beyond protein turnover [29]. Structural analyses further showed that, rather than containing multiple modular domains, URM1 adopts a ubiquitin-like β-grasp fold and retains similarities to bacterial sulfur-carrier proteins, including ThiS and MoaD [16]. Broader evolutionary studies further positioned URM1 at a transitional point between prokaryotic sulfur-transfer proteins and eukaryotic UBL modifiers [30]. This hybrid identity established URM1 as a “molecular fossil”, preserving ancestral sulfur-transfer chemistry within a eukaryotic protein-modification system [21].

This evolutionary position explains why URM1 biology cannot be fully understood through the conventional ubiquitin paradigm. URM1 shares part of the activation logic of ubiquitin-like modifiers, but its activated form also participates in sulfur transfer for cytosolic tRNA modification [19,20]. A major conceptual shift occurred when functional proteomics and biochemical studies converged, demonstrating that URM1 functions not only as a protein modifier but also as a sulfur carrier required for 2-thiolation of the wobble uridine in selected cytosolic tRNAs [19,20,31]. Thus, URM1 signaling is a bifunctional system connecting covalent protein modification, sulfur transfer, tRNA chemistry, and translational control.

### 2.2. The MOCS3/Uba4–URM1 Sulfur-Transfer Axis

The classical URM1 pathway is centered on Uba4 in yeast and its mammalian counterpart MOCS3. Mechanistically, Uba4/MOCS3 couples ATP-dependent activation of the URM1 C-terminal glycine to sulfur transfer through an adenylation domain and a rhodanese-like sulfurtransferase domain [32,33]. Upstream sulfur mobilization involves the cysteine desulfurase NFS1 and the sulfurtransferase TUM1/MPST, which contribute to sulfur transfer toward MOCS3 and thereby connect cellular sulfur metabolism to URM1 activation. Mechanistic and structural studies have further defined the formation and release of activated URM1 intermediates and the critical role of conserved catalytic cysteines in generating thiocarboxylated URM1 (URM1-COSH) [34]. Importantly, URM1-COSH is not restricted to the tRNA-modification branch. It serves as the sulfur donor for cytosolic tRNA U34 thiolation and also provides the chemical basis for thiocarboxylate-dependent protein URMylation. Notably, MOCS3 also participates in molybdenum cofactor biosynthesis by activating and transferring sulfur to MOCS2A, therefore underscoring its broader role in cellular sulfur metabolism and the potential physiological consequences of targeting this enzyme [32].

Downstream of URM1 thiocarboxylation, CTU1 and CTU2 form the principal cytosolic tRNA-thiolation machinery. Notably, URM1-dependent sulfur transfer is required for 2-thiolation of wobble uridine 34 in the selected cytosolic tRNAs [19,20]. CTU1/CTU2 act downstream of the URM1 sulfur-carrier step to incorporate sulfur into the U34 modification pathway [35]. This reaction occurs within a broader wobble uridine modification network that includes ALKBH8 and Elongator-associated enzymes, which contribute to the formation of mature U34 modifications and codon-dependent translational decoding [19,36,37,38]. Thus, the classical MOCS3/Uba4 pathway likely generates the shared thiocarboxylated URM1 intermediate that links sulfur metabolism to both tRNA modification and protein-conjugation chemistry.

Importantly, the classical URM1 activation system should not be viewed exclusively as dedicated to tRNA thiolation. Early and subsequent studies showed that URM1 can form covalent protein conjugates and that oxidative stress enhances their formation in yeast and mammalian cells [17,18]. Thus, MOCS3/Uba4-dependent URM1 activation provides a biochemical foundation for both sulfur-carrier function and stress-responsive protein URMylation, although the enzymatic requirements and substrate selectivity of mammalian protein URMylation remain incompletely resolved (Figure 1).

### 2.3. Emerging Mammalian Protein-URMylation Enzymology

In addition to thiocarboxylate-dependent protein conjugation described in yeast and reconstituted biochemical systems, a recent study proposed NAE1/UBA3 and UBE2M as E1- and E2-like components of mammalian protein URMylation under basal and oxidative stress conditions [37]. This finding is mechanistically notable because NAE1/UBA3 and UBE2M are canonical components of the NEDD8 pathway. However, this model has not yet been independently validated by other groups, and current experimental evidence does not establish that it replaces the classical MOCS3/Uba4–URM1-COSH route (Table 1). Rather, the two mechanisms may operate in parallel, overlap under specific conditions, engage distinct substrate pools, or be differentially utilized depending on cellular or tissue context, and these possibilities remain unresolved to date. In the same study, disruption of the UBE2M–DCN1 interface was found to reduce stress-induced URM1 conjugation, implicating DCN1 as a proposed E3-like cofactor or scaffold. However, direct substrate-transfer reconstitution has not yet established DCN1 as a canonical URM1 E3 [37]. Excess NEDD8 also reduced formation of putative E1~URM1 and E2~URM1 intermediates in vitro, suggesting possible competition for shared enzymatic machinery under defined biochemical conditions. This observation, however, does not demonstrate endogenous competition between NEDD8 and URM1 in intact cells. These mechanistic overlaps also complicate therapeutic interpretation. Pharmacological inhibition of NAE1/UBA3 or UBE2M can alter neddylation and cullin–RING ligase activity, making it difficult to attribute downstream anti-cancer effects specifically to protein URMylation. Accordingly, genetic perturbation, rescue experiments, direct measurement of URM1 conjugation, and parallel assessment of neddylation will be required to distinguish URM1-specific effects from broader disruption of the NEDD8 pathway [11,37].

### 2.4. Branch-Specific Regulatory Logic and UBL Cross-Talk

Available evidence indicates that the two major outputs of the URM1 signaling share upstream sulfur-transfer chemistry but are not necessarily regulated in parallel. To this end, protein URMylation is most clearly induced by acute oxidative or thiol-reactive stress, with oxidant treatment markedly increasing URM1–protein conjugate formation in yeast and mammalian cells [18,37]. In contrast, URM1-dependent tRNA U34 thiolation is dynamically influenced by nutrient availability and cellular stress state. In yeast, sulfur amino acid limitation reduces wobble uridine thiolation, thereby linking this branch to metabolic adaptation [42]. Moreover, selected stress conditions can alter tRNA thiolation in a stress- and context-dependent manner, indicating that this output is not simply coupled to the induction of protein URMylation. Together, these observations support a model in which protein URMylation and tRNA thiolation are likely chemically interconnected through URM1 activation yet can be differentially modulated by stress type, duration, metabolic state, and cellular context. Whether the two branches compete for, or are coordinately regulated through, a common pool of activated URM1 remains unresolved, particularly in mammalian cancer cells.

Recent evidence also reveals a mechanistic intersection between URM1 and the NEDD8 pathway. NAE1/UBA3 and UBE2M are established components of the neddylation machinery and have recently been implicated in mammalian protein URMylation [11,36]. In vitro, excess NEDD8 reduced the formation of putative URM1-charging intermediates involving this shared E1/E2 machinery, suggesting competition for enzyme engagement under defined biochemical conditions [37]. However, these findings do not establish global competition between endogenous NEDD8 and URM1 in intact cells, where relative abundance, compartmentalization, substrate availability, and stress-dependent regulation may influence pathway selection. By contrast, comparable sharing of a physiological E1/E2 cascade between URM1 and other UBL systems, such as SUMO, has not been demonstrated. Thus, NEDD8–URM1 cross-talk represents an experimentally supported area of pathway convergence, whereas broader interactions between URM1 and other UBL systems remain largely unexplored to date.

**Figure 2:**
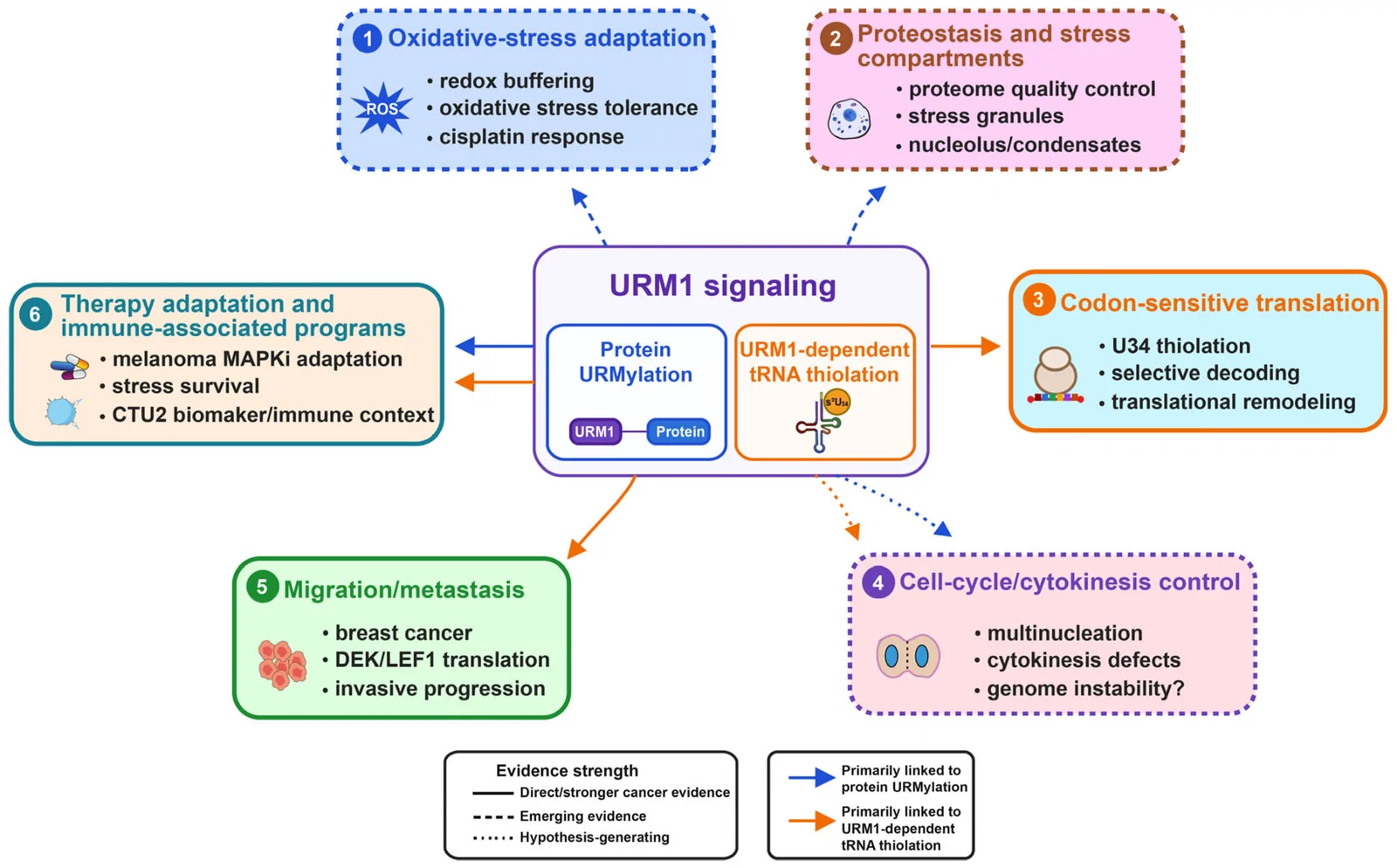
Mechanistic links between URM1 signaling and cancer biology. URM1 signaling, encompassing protein URMylation and URM1-dependent tRNA U34 thiolation, is positioned at the center and linked to six cancer-relevant biological processes: oxidative-stress adaptation, proteostasis and stress compartments, codon-sensitive translation, cell cycle/cytokinesis control, migration/metastasis, and therapy adaptation and immune-associated programs. These modules summarize the current evidence linking URM1-associated pathways to cancer phenotypes, ranging from mechanistically supported relationships to emerging observations and hypotheses that require further tumor-specific validation. Evidence strength is indicated by line style as defined in the figure key.

These biochemical distinctions provide the framework for interpreting the cancer-associated phenotypes discussed below. Protein URMylation is most directly linked to redox-responsive protein remodeling and cellular stress adaptation, whereas URM1-dependent tRNA U34 thiolation supports codon-sensitive translation and proteome maintenance and has been implicated in metastatic and therapy-adapted tumor states. In contrast, phenotypes observed after perturbation of shared upstream components, such as URM1 or MOCS3, cannot be assigned to either branch without branch-specific biochemical measurements or evidence from functional rescue experiments. Section 3 therefore evaluates cancer-associated evidence according to both biological phenotype and the degree of branch-specific mechanistic support (Figure 2).

## 3. Functional Outputs and Cancer-Relevant States of URM1 Signaling

The molecular architecture described above generates two chemically linked yet biologically distinct outputs. Covalent protein URMylation functions primarily as a stress-responsive protein-modification process, whereas URM1-dependent tRNA thiolation links sulfur chemistry to codon-sensitive translational control. In cancer, these outputs are unlikely to define a single linear oncogenic pathway; rather, available evidence suggests that URM1-associated biology becomes most relevant in tumor states that depend on redox buffering, selective translation, proteome management, and metabolic adaptation. The strength of evidence varies across different cancer contexts. Hepatocellular carcinoma provides the most direct URM1-focused cancer data, whereas breast cancer and melanoma support a putative role for the URM1-related U34 tRNA-modification network in metastasis and therapy adaptation (Table 1). By contrast, links to cytokinesis, genome integrity, and immune-associated biomarkers remain more hypothesis-generating at this stage (Figure 2).

### 3.1. Protein URMylation, Redox Adaptation, and Hepatocellular Carcinoma

Protein URMylation represents the covalent protein-modification output of the URM1 system (Table 2). Its best-established regulatory feature is stress inducibility: oxidative or thiol-reactive stress markedly increases URM1–protein conjugate formation in yeast and mammalian cells [18]. The yeast peroxiredoxin Ahp1 remains the best-characterized endogenous substrate, providing early mechanistic evidence that URM1 conjugation contributes to oxidant-stress tolerance [17]. In mammalian cells, oxidative stress-induced covalent URM1 conjugates have been reported for MOCS3, ATPBD3/CTU1, CTU2, USP15, and CAS/CSE1L (Table 2) [18], although their endogenous acceptor sites and modification-dependent functions remain incompletely defined. Complementary biochemical studies further showed that thiocarboxylated human URM1 can be conjugated to PRDX5 (the human homolog of yeast Ahp1), GAPDH, SARS1, and PKM2 under oxidative conditions, with LC-MS/MS identifying multiple Lys-, Ser-, and Thr-linked acceptor sites [43]. These findings establish redox-responsive protein-conjugation chemistry but do not yet define an endogenous, site-resolved mammalian URM1 substrate whose modification is causally required for a tumor phenotype.

This stress-responsive behavior provides a biological rationale for examining protein URMylation in tumors exposed to high oxidative pressure. Cancer cells frequently experience elevated reactive oxygen species as a consequence of oncogenic signaling, metabolic reprogramming, mitochondrial dysfunction, and anticancer therapy; although moderate ROS levels can support signaling and adaptation, excessive oxidative stress damages macromolecules and can trigger cell death [6,44]. Protein URMylation could therefore contribute to tumor fitness by modifying stress-sensitive proteins during redox challenge. However, this possibility should be distinguished from an established cancer mechanism, because direct evidence linking modification of a specific endogenous mammalian substrate to redox adaptation or tumor growth remains limited.

Hepatocellular carcinoma (HCC) currently provides one of the most direct cancer-focused contexts in which URM1-associated phenotypes have been examined, although the available primary evidence remains limited and mechanistically heterogeneous. Cheng and colleagues reported elevated URM1 expression in HCC specimens and associations between higher URM1 abundance and adverse clinical features. In liver cancer models, URM1 knockdown reduced proliferation and migration, increased apoptosis, and was accompanied by altered JNK-related signaling [39]. These findings support a functional requirement for the integrated URM1 system in these models, but they do not identify the responsible biochemical branch, as depletion of URM1 simultaneously disrupts both protein URMylation and URM1-dependent tRNA U34 thiolation.

A mechanistically distinct study by Chakraborty and colleagues implicated the proposed NAE1/UBA3–UBE2M protein-URMylation machinery in oxidative stress tolerance and cisplatin response in liver cancer cells [37]. This work provides a more direct link between protein URMylation and stress adaptation in cancer. Nevertheless, the pathway has not yet been independently validated, and interpretation is complicated by the canonical roles of NAE1/UBA3 and UBE2M in neddylation. Thus, altered NEDD8 signaling remains an important potential contributor to phenotypes produced by perturbation of this machinery.

Taken together, these studies identify HCC as a relevant model for investigating URM1-associated redox adaptation, but it warrants additional independent validation to better understand URM1-driven oncogenic mechanisms. Establishing such a mechanism will require endogenous substrate identification and site-specific validation, parallel measurement of protein URMylation and tRNA U34 thiolation, branch-informative genetic rescue, and, where the NAE1/UBA3–UBE2M pathway is examined, orthogonal assessment of canonical neddylation.

#### 3.1.1. Mammalian URMylome and Substrate-Specificity Challenges

Defining bona fide mammalian URM1 substrates remains a major technical bottleneck. Cellular studies have identified stress-induced covalent conjugates, whereas reconstituted systems have mapped URM1-derived peptide remnants to lysine, serine, and threonine residues and linked conjugation to nearby redox-active cysteines and cysteine persulfidation [18,43]. These validation levels should not be conflated: a protein that can be conjugated in vitro is not automatically an endogenous tumor substrate, and an affinity-enriched protein is not necessarily covalently URMylated.

Several features complicate endogenous URMylation mapping, including stress dependence and potentially low stoichiometry, redox-sensitive intermediates, noncanonical acceptor residues, concurrent persulfidation, and the absence of a widely validated site-specific anti-URMylation reagent. A rigorous workflow should therefore combine stress-conditioned denaturing URM1 enrichment or endogenous tagging, quantitative LC-MS/MS with URM1-remnant-aware searches, orthogonal activity-based probes, site-directed mutagenesis of candidate acceptor residues and redox-active cysteines, and genetic rescue of the relevant URM1-pathway component. Parallel measurement of tRNA U34 thiolation is important when shared upstream components are perturbed, to prevent substrate phenotypes from being misattributed to indirect translational effects.

The strongest direct cancer-focused evidence for URM1 currently comes from hepatocellular carcinoma (HCC), but the literature remains limited. Cheng and colleagues reported increased URM1 expression in HCC tissues and associated high URM1 expression with an unfavorable prognosis; URM1 knockdown reduced proliferation and migration, promoted apoptosis, and altered JNK-related signaling in liver cancer cell models [39]. A mechanistically distinct 2026 study linked NAE1/UBA3–UBE2M-dependent protein URMylation to oxidative stress protection and cisplatin sensitivity in liver cancer cells [37]. These studies are complementary but do not establish a single replicated URM1-driven HCC mechanism. In particular, the Cheng et al. depletion phenotype is branch-unresolved, whereas the NAE1/UBA3 study measures protein URMylation but may be confounded by the canonical neddylation functions of the same enzymes. Thus, HCC is a priority context for mechanistic validation, rather than evidence that URM1 is likely a universal liver-cancer oncogenic driver.

### 3.2. URM1-Dependent tRNA Thiolation, Codon-Sensitive Translation, and Tumor-State Plasticity

The second major output of URM1 signaling is tRNA thiolation, which links URM1 sulfur chemistry to translational control. URM1-dependent U34 thiolation does not simply increase or decrease global protein synthesis. Instead, it improves decoding efficiency and fidelity for selected codon families, thereby connecting tRNA chemistry to codon-sensitive translation [19,31]. Changes in U34 thiolation may therefore preferentially affect transcripts whose translation depends on codons decoded by U34-modified tRNAs, reshaping specific protein networks without necessarily producing a large change in bulk protein synthesis (Figure 2).

This selectivity is relevant to cancer because malignant phenotypes can depend on selective translational programs rather than on global increases in protein synthesis [45,46]. Metastatic or therapy-adapted cancer cells may rely on selective translational programs that promote invasion, phenotypic plasticity, metabolic adaptation, and survival under therapeutic stress [26,38,45,46,47]. Breast cancer provides one of the clearest examples. To this end, the broader U34-modification network, including ELP3 and the partner enzymes CTU1 and CTU2, is upregulated in invasive disease and supports metastatic progression (Table 3) [38]. Mechanistically, this pathway promotes translation of pro-metastatic regulators, linking wobble uridine modification to defined metastatic outputs rather than to nonspecific growth effects [38]. U34-modifying enzymes have been linked to tumor-cell survival and targeted-therapy resistance through codon-selective translation of specific mRNAs [47]. These studies do not establish URM1 itself as a universal mediator of metastasis or drug resistance. Rather, they indicate that the URM1-related tRNA-thiolation network can become functionally relevant when tumor cells depend on codon-sensitive translation during invasion, metastatic progression, or treatment-induced plasticity.

### 3.3. Proteostasis-Related Stress Responses, Condensate Hypothesis, and Metabolic Control of Pathway Activity

Beyond its established roles in stress-responsive protein conjugation and tRNA-dependent translational control, URM1 signaling may intersect with proteostasis-related stress responses, although the strength of evidence varies substantially across biological contexts. In yeast, Uba4 and URM1 participate in stress-regulated phase separation, and localized URMylation of proteins has been linked to the recruitment or deposition of selected proteins into stress-associated assemblies, including stress granules and the nucleolus [41,48]. These studies provide a mechanistic precedent for an interaction between protein URMylation and condensate biology in yeast. However, a comparable URM1-dependent mechanism has not yet been demonstrated in mammalian cells or tumors. Condensate regulation should therefore be considered a hypothesis-generating extension of yeast biology rather than an established mammalian or cancer-specific output of URM1 signaling. Future studies will need to determine whether stress-induced mammalian protein URMylation alters the recruitment, retention, dynamics, or recovery of specific proteins within stress granules, nucleoli, or other tumor-associated condensates [49,50,51].

The sulfur dependence of URM1 chemistry provides a separate metabolic dimension that may influence pathway activity. Formation of thiocarboxylated URM1 and URM1-dependent tRNA U34 thiolation requires cellular sulfur mobilization, suggesting that pathway output may vary with nutrient availability, redox state, and sulfur-metabolic flux rather than with expression of URM1-pathway components alone [19,32]. This distinction may be particularly relevant in cancer, where cysteine uptake, glutathione synthesis, methionine metabolism, and other sulfur-dependent processes are frequently remodeled during tumor growth and stress adaptation [52,53,54]. However, such metabolic remodeling does not by itself establish increased protein URMylation or tRNA U34 thiolation. Direct biochemical measurements are therefore required to determine whether altered sulfur metabolism translates into enhanced URM1-pathway activity in a given tumor context.

These considerations support a dynamic rather than expression-defined view of URM1 signaling. As such, tumor cells with modest basal abundance of URM1-pathway components may acquire pathway dependence under oxidative stress, nutrient limitation, or treatment-induced stress. In contrast, high expression alone does not establish active protein URMylation, sustained tRNA U34 thiolation, or functional dependency. Accordingly, future studies should integrate measurements of the sulfur metabolic state with branch-specific readouts of URM1 protein conjugation and tRNA U34 thiolation to determine when URM1 signaling becomes functionally limiting in cancer.

### 3.4. Branch-Resolved Interpretation and Current Boundaries

Because URM1 signaling produces more than one biochemical output, phenotypes resulting from pathway perturbation require branch-resolved interpretation [32,35,37]. Perturbing shared upstream components may affect both protein URMylation and tRNA thiolation, whereas disruption of branch-restricted components is more informative for assigning functional output (Figure 2). In cancer studies, this distinction is essential: URM1-associated phenotypes may reflect covalent protein modification, altered tRNA decoding, disrupted sulfur-transfer chemistry, or combined stress responses. For this reason, the term “protein URMylation” should be reserved for contexts in which covalent URM1 conjugation is directly demonstrated, whereas “URM1 signaling” is more appropriate when multiple pathway outputs may contribute.

Several cancer-relevant observations remain unresolved regarding branch assignment. Depletion of URM1 in HeLa cells causes pronounced defects in cytokinesis and multinucleation [20]. However, because URM1 functions as both a protein modifier and a sulfur carrier required for tRNA U34 thiolation, depletion of URM1 simultaneously perturbs both outputs, thereby failing to establish which branch is responsible for the phenotype. Accordingly, these findings should not be interpreted as direct evidence that protein URMylation regulates cytokinesis. Resolving this question will require branch-informative perturbation of the tRNA-thiolation machinery, parallel quantification of protein URM1 conjugates and tRNA U34 thiolation, and branch-specific rescue experiments. If protein URMylation is implicated, endogenous substrates whose covalent modification is functionally required should then be identified and validated. Thus, potential links between URM1 signaling, cytokinesis failure, tetraploidy, aneuploidy, and chromosomal instability in cancer remain mechanistically plausible but currently hypothesis-generating [55,56,57].

Expression-based and pan-cancer analyses provide an additional, but distinct, layer of experimental evidence. CTU1 and CTU2 are core components of the tRNA U34-thiolation machinery, and CTU2 expression has been associated with prognosis and immunotherapy-related clinical features in pan-cancer analyses [40,58]. However, elevated CTU1 or CTU2 expression should not be considered a direct surrogate for tRNA-thiolation activity or functional pathway dependence. Such associations may also reflect tumor lineage, proliferative state, copy-number alterations, cellular stress, stromal composition, or immune-cell content. Functional evaluation in clinical specimens should therefore incorporate more proximal biochemical readouts. These may include quantitative LC-MS/MS measurement of modified tRNA nucleosides, tRNA-specific thiolation assays, and, where feasible, codon-aware translational profiling. Ideally, these measurements should be integrated with CTU1/CTU2 expression and compared between tumor and matched normal tissues. CTU1/CTU2 abundance should therefore be viewed as a complementary biomarker candidate rather than a stand-alone indicator of URM1-dependent tRNA-thiolation activity.

## 4. Therapeutic Opportunities in Precision Oncology

**Figure 3:**
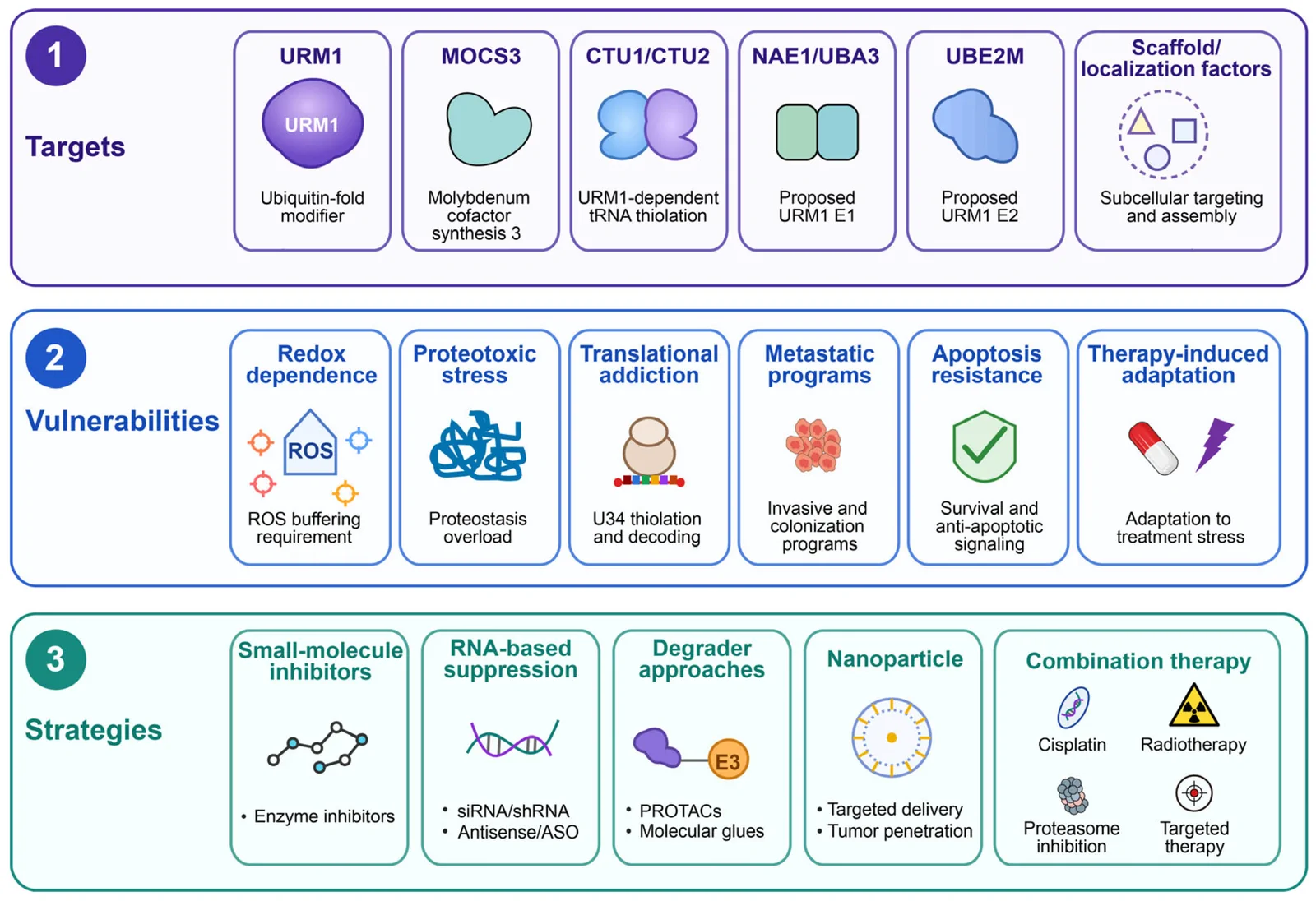
Therapeutic targeting framework for URM1-associated cancer vulnerabilities. The schematic is organized into three conceptual layers. The upper layer summarizes candidate molecular nodes within the URM1 system, including URM1, MOCS3/Uba4, CTU1/CTU2, NAE1/UBA3, UBE2M, and potential scaffold or localization factors. The middle layer highlights cancer-associated vulnerabilities potentially linked to these nodes, including redox dependence, proteotoxic stress, translational addiction, metastatic programs, apoptosis resistance, and therapy-induced adaptation. The lower layer outlines potential intervention strategies, including small-molecule inhibitors, RNA-based suppression, degrader approaches, nanoparticle-based delivery, and combination therapies with cisplatin, radiotherapy, proteasome inhibition, or targeted therapy. These therapeutic concepts span different levels of experimental support and therefore represent a framework for further validation rather than clinically established URM1-directed interventions.

Therapeutic development targeting the URM1 signaling remains in its early stages, and Figure 3 is intended as an evidence-stratified roadmap rather than a catalog of validated therapies. Moreover, no selective clinical inhibitor of URM1, MOCS3, CTU1, CTU2, or URM1-specific protein conjugation has been established to date. The only direct cancer-linked pharmacological evidence currently comes from perturbation of the shared NAE1/UBA3–UBE2M/DCN1 machinery [37]; even there, canonical neddylation creates an important specificity confounder. Therefore, other modalities shown in Figure 3 should be considered as future tool-development concepts.

### 4.1. Therapeutic Entry Points and Tool-Development Challenges

Several components of the URM1 system are conceptually druggable, but their shared physiological functions impose substantial constraints on therapeutic selectivity and safety. MOCS3 is particularly important in this regard because it supports URM1 thiocarboxylation and URM1-dependent tRNA U34 thiolation while also participating in molybdenum cofactor biosynthesis through sulfur transfer to MOCS2A [32]. Systemic MOCS3 inhibition could therefore disrupt both translational homeostasis and molybdoenzyme-dependent metabolism in normal tissues, rather than selectively suppressing a tumor-specific URM1 dependency. CTU1/CTU2 inhibition raises a related concern, as U34 thiolation contributes to efficient and accurate translation in both normal proliferating and stress-exposed cells. Thus, branch-selective targeting should be viewed as a future therapeutic objective that requires experimental demonstration rather than an established property of the pathway.

Defining a therapeutic window will require early comparison of tumor and matched normal models, including primary cells and organoids where feasible. These studies should incorporate direct pharmacodynamic measurements of protein URMylation, tRNA U34 thiolation, and relevant physiological outputs. Potential risk-mitigation strategies include partial or intermittent pathway suppression, tumor- or tissue-selective delivery, biomarker-guided patient selection, and combination regimens that may permit lower pathway-inhibitor exposure. In the longer term, targeting branch-restricted protein–protein interactions or substrate-specific regulatory interfaces may offer greater selectivity than inhibiting shared upstream sulfur-transfer components. Such strategies will likely depend on identifying and validating the relevant endogenous enzymes, interaction surfaces, and cancer-specific substrates.

The recently proposed NAE1/UBA3–UBE2M route offers a more pharmacologically accessible entry point but introduces an additional specificity challenge. NAE1/UBA3 and UBE2M are canonical components of the NEDD8 pathway, and pharmacological perturbation of these enzymes can alter neddylation and cullin–RING ligase activity. Pevonedistat, for example, was developed as an inhibitor of NEDD8 activation; its anticancer effects therefore cannot be attributed specifically to protein URMylation without orthogonal genetic and biochemical evidence [37,59,60]. Similarly, currently available NAE1/UBA3-, UBE2M-, or DCN1-directed compounds should be regarded primarily as mechanistic probes for testing the emerging mammalian protein-URMylation model rather than as selective URMylation therapeutics. URM1-specific effects will require direct measurement of URM1 conjugation together with parallel assessment of canonical neddylation.

Future tool development may enable more selective interrogation of URM1 signaling. To this end, chemically induced proximity, engineered recruitment systems, or other substrate- and interface-directed approaches could, in principle, provide greater control over individual URM1-dependent processes than broad pathway inhibition [61]. However, these strategies remain conceptual in the URM1 field and should not yet be considered established therapeutic modalities. Their development will depend on a more complete definition of mammalian protein-URMylation enzymology, endogenous substrate specificity, branch-selective biomarkers, and tumor–normal dependencies.

### 4.2. Stress-Conditioned Combination Strategies

The most defensible near-term therapeutic concept is stress-conditioned sensitization, but the evidence should be separated from extrapolation. Direct support currently exists for enhanced cisplatin killing after perturbation of the NAE1/UBA3–UBE2M/DCN1-associated protein-URMylation pathway in liver cancer cells [37]. Combinations with radiotherapy, ferroptosis induction, proteasome inhibition, or other targeted therapies remain hypotheses to be tested rather than established URM1-directed regimens. In each case, the central question is whether pathway perturbation shifts a defined stress-adaptation program toward cell death without producing unacceptable injury in normal tissues.

This strategy will require mechanism-based assessment of response rather than endpoint viability alone. Combination studies should determine whether pathway perturbation changes URM1-conjugate formation, tRNA U34 thiolation, oxidative damage, translational output, apoptotic or ferroptotic response, and post-treatment recovery. Candidate responsive tumors may include those with high basal oxidative burden, limited antioxidant reserve, inducible URM1 conjugation, sulfur metabolic dependence, or reliance on codon-sensitive stress-response translation. Resistance may arise through glutathione remodeling, compensatory redox pathways, alternative tRNA-modification programs, or broader stress-response rewiring. URM1-directed combinations should therefore be developed as precision stress-sensitization strategies rather than as conventional cytotoxic regimens.

### 4.3. Biomarker-Guided Patient Selection and Delivery Strategy

A precision-oncology approach to URM1 signaling will require biomarkers that reflect pathway activity and functional dependence rather than expression alone. Elevated levels of URM1, MOCS3, CTU1, or CTU2 do not necessarily indicate increased protein URMylation, sustained tRNA U34 thiolation, or tumor dependence on either branch. More informative pharmacodynamic and patient-selection markers may include stress-inducible URM1-conjugate profiles, quantitative LC-MS/MS measurements of modified tRNA nucleosides, and tRNA-specific thiolation assays. Additional indicators may include codon-biased translational outputs, redox and sulfur metabolic states, and pathway-specific responses to genetic or pharmacological perturbation (Figure 3). Whenever feasible, these measurements should be performed on tumor samples alongside matched normal tissues and integrated with functional dependency data to determine whether a therapeutically exploitable differential exists.

Such tumor–normal selectivity will be particularly important because systemic inhibition of shared components of the URM1 pathway may disrupt essential physiological functions. MOCS3, for example, contributes not only to URM1 activation and tRNA U34 thiolation but also to molybdenum cofactor biosynthesis, raising the possibility of on-target effects on translational homeostasis and molybdoenzyme-dependent metabolism in normal tissues. Similarly, sustained disruption of the tRNA-thiolation branch may impair stress adaptation and translational fidelity in proliferating or metabolically active cells. These considerations argue for cautious development of URM1-directed strategies and favor approaches that minimize systemic pathway suppression. Such approaches may include tumor- or tissue-selective delivery, partial or intermittent dosing, and, where mechanistically feasible, targeting branch-restricted protein–protein interactions rather than shared upstream enzymes. Biomarker-guided combination regimens may also permit lower pathway-inhibitor exposure while retaining antitumor activity. Overall, therapeutic translation of URM1 signaling will require both a branch-specific pharmacodynamic response and a sufficient therapeutic window between tumor and normal tissues. This goal is more likely to be achieved through selective, context-dependent targeting than through indiscriminate pathway inhibition as a universal monotherapy.

## 5. Future Directions and Methodological Priorities

The next stage of URM1 research in cancer should shift from expression-based associations to activity- and mechanism-resolved pathway analysis. A major priority should be the systematic identification of the cancer-associated URMylome under physiologically relevant stress conditions. Because protein URMylation is stress-responsive, functionally important substrates may not be constitutively modified under basal culture conditions but may emerge during oxidative stress, chemotherapy, hypoxia, nutrient limitation, or proteotoxic challenge [18]. Future studies should combine stress-conditioned substrate mapping, modification-site identification, and functional validation. These approaches will help determine whether URMylation alters substrate localization, redox sensitivity, enzymatic activity, condensate association, interaction networks, or stress recovery. These studies should also distinguish protein URMylation from URM1-dependent tRNA thiolation by pairing pathway perturbation with direct biochemical readouts. These may include URM1-conjugate detection, quantitative measurement of tRNA U34 thiolation, substrate-level proteomics, and codon-aware ribosome profiling.

Comparative analysis of ubiquitin and representative ubiquitin-like modifier systems highlights an additional gap in the URM1 field (Table 4). Most well-characterized UBL pathways are organized around defined E1, E2, E3, and deconjugating enzyme modules, which provide regulatory specificity and pharmacological entry points. By contrast, URM1 signaling remains enzymatically incomplete. Uba4 in yeast and MOCS3 in mammals constitute the classical URM1-activating machinery, whereas NAE1/UBA3 and UBE2M have recently been proposed to support mammalian protein URMylation, particularly under oxidative stress [32,44]. However, canonical URM1-specific E3 ligases and de-URMylating proteases have not yet been clearly established. Identifying such enzymes should be a major priority for future work, because they may determine substrate specificity, pathway reversibility, stress responsiveness, and tissue-selective URM1 signaling. In particular, discovery of URM1-directed deconjugating proteases would create new opportunities to develop selective inhibitors that stabilize URM1-conjugated substrates or modulate stress-induced URMylation in defined tumor contexts.

A second unresolved issue is whether different URM1-activating routes operate in distinct tissues, tumor types, or stress states. The classical MOCS3/Uba4-dependent pathway is closely linked to sulfur transfer and tRNA thiolation, whereas the emerging NAE1/UBA3–UBE2M model suggests that mammalian protein URMylation may also engage enzymes classically assigned to the NEDD8 pathway [32,44]. These routes should not be assumed to be interchangeable. Their relative contribution may vary according to lineage, metabolic state, oxidative burden, subcellular localization, substrate availability, or therapeutic stress. Future studies should therefore compare MOCS3-dependent and NAE1/UBA3-dependent URM1 outputs across tumor types and normal tissues using branch-selective perturbation, rescue experiments, substrate-level proteomics, and direct measurements of tRNA U34 thiolation. Such work will be essential for determining whether URM1 signaling is governed by a universal enzymatic architecture or by tissue- and tumor-state-specific activation logic. Consistent with this need, curated mouse genetics data indicate that homozygous URM1 deficiency causes developmental delay, placentation defects, and embryonic lethality during organogenesis. By contrast, systematic in vivo phenotyping of Mocs3/Uba4 deficiency remains limited, supporting the use of future conditional or tissue-specific URM1 and Mocs3/Uba4 models to distinguish developmental, tissue-homeostatic, and tumor-context requirements.

Another priority is to define the tumor states in which URM1 signaling becomes functionally limiting. The most relevant question is not whether URM1 is broadly oncogenic but when its stress-responsive and translational outputs become necessary for tumor-cell fitness. Hepatocellular carcinoma remains a key model for URM1-focused studies, whereas breast cancer and melanoma provide complementary settings for investigating the tRNA-thiolation branch in metastasis and therapy adaptation [26,38,39,47]. Additional candidate settings may include tumors with high oxidative burden, proteotoxic stress, sulfur-metabolism rewiring, aneuploidy-associated translational pressure, or therapy-induced plasticity. Stress-conditioned genetic screens, patient-derived organoids, patient-derived xenografts, and ex vivo tumor-slice models will be important for determining whether URM1-pathway dependence is revealed only under specific microenvironmental or therapeutic pressures [62,63].

Finally, therapeutic translation will require a rigorous evidence framework that integrates pathway activity, selectivity, and safety. Claims of URM1-pathway dependence should include demonstration of pathway engagement in the relevant tumor model, as well as branch-specific biochemical validation. Genetic or pharmacological perturbation should be supported by appropriate rescue experiments and confirmation in at least one physiologically relevant system. Combination studies should measure not only endpoint viability but also protein URMylation, tRNA thiolation, redox damage, translational remodeling, cell-death mode, and post-treatment recovery. Safety assessment should be incorporated early, because URM1 signaling may also protect normal tissues under physiological stress. Establishing these standards will help the field move beyond descriptive correlations and determine whether URM1 signaling represents a context-specific cancer vulnerability with genuine translational potential.

## 6. Conclusions

URMylation represents a chemically distinctive ubiquitin-like system that links protein conjugation, sulfur transfer, tRNA thiolation, translational control, and stress adaptation. This dual-output architecture makes URM1 signaling conceptually different from canonical ubiquitination and other more extensively characterized UBL pathways. In cancer biology, its relevance is unlikely to be explained by a single dominant oncogenic substrate or a uniform growth-promoting function. Instead, current evidence supports a more context-dependent model in which URM1-associated pathways may help tumor cells maintain redox balance, proteome quality, translational fidelity, and survival under metabolic or therapeutic stress.

The strongest cancer evidence spans several distinct contexts. These include URM1-associated phenotypes in hepatocellular carcinoma, U34 tRNA-modification programs in metastasis and therapy adaptation, and stress-induced protein URMylation linked to oxidative-stress protection and cisplatin response [26,37,38,39,47]. These findings suggest that URM1 signaling may become most relevant in tumor states characterized by oxidative burden, sulfur-metabolic rewiring, codon-sensitive translational demand, or treatment-induced plasticity. However, they do not yet establish URMylation as a mature or universal therapeutic target. Future progress will depend on branch-resolved assays, activity-based biomarkers, validated substrate maps, and tumor-context-specific models that can distinguish protein URMylation from URM1-dependent tRNA thiolation. If such studies define selective tumor-state dependencies, URM1 signaling could emerge as a valuable stress-adaptation pathway for biomarker-guided precision cancer therapy.

## Figures and Tables

**Table 1:** Branch-resolved summary of cancer-associated evidence for URM1 signaling.

Cancer Context	Phenotype/Observation	Supported Branch (Protein URMylation vs. tRNA Thiolation)	Evidence Level	References
Hepatocellular carcinoma	URM1 expression; growth, migration, apoptosis and JNK-related phenotypes	Branch unresolved	Clinical association + genetic/functional	[39]
Liver cancer cells	NAE1/UBA3–UBE2M/DCN1 perturbation reduces protein URMylation and sensitizes cells to cisplatin	Protein URMylation	Biochemical + genetic + pharmacological; single study	[37]
Breast cancer	ELP3/CTU1/CTU2-dependent U34 modification supports DEK/LEF1-linked metastasis	tRNA thiolation	Mechanistic + functional	[38]
HeLa cytokinesis	URM1 depletion causes cytokinesis defects and multinucleation	Branch unresolved	Cell-biologic functional	[20]
Pan-cancer CTU2 analyses	Prognostic and immune-microenvironment associations	tRNA thiolation component	Expression/clinical association	[40]
Biomolecular condensates	Stress-dependent URM1/Uba4-dependent condensate organization	Protein URMylation-related	Strong yeast mechanism; cancer hypothesis	[41]

**Table 2:** Experimentally supported mammalian protein-URMylation substrates and current cancer relevance.

Substrate	Experimental Context	Validation Level	URMylation Sites	References
MOCS3	Human cells under oxidative stress	Cellular covalent conjugation	Not site-resolved; lysine-directed	[18]
ATPBD3/CTU1	Human cells under oxidative stress	Cellular covalent conjugation	Not site-resolved; lysine-directed	[18]
CTU2	Human cells under oxidative stress	Cellular covalent conjugation	Not site-resolved; lysine-directed	[18]
USP15	Human cells under oxidative stress	Stress-associated cellular substrate	Not site-resolved; lysine-directed	[18]
CAS/CSE1L	Human cells under oxidative stress	Cellular covalent substrate	Not mapped	[18]
PRDX5	Purified human proteins + URM1-COSH + oxidant	In vitro covalent conjugation + site-level MS	K116, K118, K191, T203	[43]
GAPDH	Purified human proteins + URM1-COSH + oxidant	In vitro covalent conjugation + site-level MS	K219, K227, K259/K260, K263	[43]
SARS1	Purified human proteins + URM1-COSH + oxidant	In vitro covalent conjugation + site-level MS	K28, K57, K62, K67, K69, K123, K154, K197, S298, T299, K323, S366, K455	[43]
PKM2	Purified human proteins + URM1-COSH + oxidant	In vitro covalent conjugation + site-level MS	S3, K4, T115, K126, K136/K137, T140, K142, K163, K167, S173, K174, K271, K306, S347, K368, K423, K476, K499, K505/K506	[43]

**Table 3:** Cancer-associated translational effectors linked to the U34 tRNA-modification network.

Cancer Context	U34-Pathway Components	Translation-Sensitive Protein	Downstream Consequence	References
Breast cancer metastasis	ELP3, CTU1/CTU2	DEK	Supports IRES-dependent LEF1 translation and invasive/metastatic behavior	[38]
BRAF-driven melanoma/MAPK-targeted therapy resistance	ELP3 and U34-modifying enzymes including CTU1/CTU2	HIF1A	Linked to HIF1A-dependent glycolytic adaptation and resistance to MAPK-targeted therapy	[26]

**Table 4:** Comparison of ubiquitin and representative UBL modifier systems with their E1/activation, E2, E3, and deconjugation machinery.

Ubiquitin/UBLs	E1	E2	E3	UBL Proteases
Ubiquitin	UBA1/UBA6	Multiple	Multiple	DUBs
SUMO1/2/3	SAE1/UBA2	UBC9/UBE2I	Multiple	SENPs
ISG15	UBA7	UBCH6/8	Multiple	USP18
NEDD8	NAE1/UBA3	UBC12/UBE2F	RBX1/2	CSN5/DEN1
UFM1	UBA5	UFC1	UFL1	UFSP1/2
URM1	Uba4 (yeast)/MOCS3 (mammals); NAE1/UBA3 (proposed, requires validation)	UBE2M (proposed, needs validation)	DCN1 (proposed cofactor, needs validation)	None established yet

## Data Availability

No new data were created or analyzed in this study. Data sharing is not applicable to this article.

## Abbreviations

The following abbreviations are used in this manuscript:

ATPBD3	ATP-binding domain-containing protein 3
BRAF	B-Raf proto-oncogene, serine/threonine kinase
CSE1L	Chromosome segregation 1 like
CTU1	Cytosolic thiouridylase subunit 1
CTU2	Cytosolic thiouridylase subunit 2
DCN1	Defective in cullin neddylation 1
DEK	DEK proto-oncogene
ELP3	Elongator complex protein 3
GAPDH	Glyceraldehyde-3-phosphate dehydrogenase
HCC	Hepatocellular carcinoma
HIF1A	Hypoxia-inducible factor 1 subunit alpha
IRES	Internal ribosome entry site
ISG15	Interferon-stimulated gene 15
JNK	c-Jun N-terminal kinase
LC-MS/MS	Liquid chromatography–tandem mass spectrometry
LEF1	Lymphoid enhancer-binding factor 1
MAPK	Mitogen-activated protein kinase
MOCS2A	Molybdenum cofactor synthesis protein 2A
MOCS3	Molybdenum cofactor sulfurase C-terminal domain-containing protein 3
NAE1	NEDD8-activating enzyme (NAE) E1 regulatory subunit
PKM2	Pyruvate kinase M2
PRDX5	Peroxiredoxin 5
PTM	Post-translational modification
ROS	Reactive oxygen species
SAE1	SUMO1 activating enzyme subunit 1
SARS1	Seryl-tRNA synthetase 1
SUMO	Small ubiquitin-like modifier
U34	Uridine at wobble position 34 of tRNA
UBA1	Ubiquitin-like modifier activating enzyme 1
UBA2	Ubiquitin-like modifier activating enzyme 2
UBA3	Ubiquitin-like modifier activating enzyme 3
UBA4	Ubiquitin-like modifier activating enzyme 4
UBA5	Ubiquitin-like modifier activating enzyme 5
UBA6	Ubiquitin-like modifier activating enzyme 6
UBA7	Ubiquitin-like modifier activating enzyme 7
UBCH6/8	Ubiquitin-conjugating enzymes E2E 1/E2 L6
UBC9	SUMO-conjugating enzyme UBC9 (UBE2I)
UBC12	NEDD8-conjugating enzyme UBC12 (UBE2M)
UBE2F	Ubiquitin-conjugating enzyme E2 F
UBE2I	Ubiquitin-conjugating enzyme E2 I
UBE2M	Ubiquitin-conjugating enzyme E2 M
UBL	Ubiquitin-like
UFC1	UFM1-specific E2 conjugating enzyme 1
UFM1	Ubiquitin-fold modifier 1
URM1	Ubiquitin-related modifier 1
USP15	Ubiquitin-specific peptidase 15
